# Electrical stimulation improved cognitive deficits associated with traumatic brain injury in rats

**DOI:** 10.1002/brb3.667

**Published:** 2017-10-05

**Authors:** Zhi‐tong Zheng, Xin‐long Dong, Ya‐dan Li, Wei‐wei Gao, Yuan Zhou, Rong‐cai Jiang, Shu‐yuan Yue, Zi‐wei Zhou, Jian‐ning Zhang

**Affiliations:** ^1^ Department of Neurosurgery Tianjin Neurological Institute Tianjin Medical University General Hospital Tianjin China; ^2^ Key Laboratory of Post‐trauma Neuro‐repair and Regeneration in Central Nervous System Ministry of Education Tianjin China; ^3^ Tianjin Key Laboratory of Injuries, Variations and Regeneration of Nervous System Tianjin China; ^4^ Intensive Care Units Tianjin Huanhu Hospital Tianjin China

**Keywords:** angiogenesis, cognitive deficit, electrical stimulation, endothelial progenitor cell, traumatic brain injury

## Abstract

**Introduction:**

Cognitive deficits associated with traumatic brain injury (TBI) reduce patient quality of life. However, to date, there have been no effective treatments for TBI‐associated cognitive deficits. In this study, we aimed to determine whether electrical stimulation (ES) improves cognitive deficits in TBI rats.

**Methods:**

Rats were randomly divided into three groups: the Sham control group, electrical stimulation group (ES group), and No electrical stimulation control group (N‐ES group). Following fluid percussion injury, the rats in the ES group received ES treatment for 3 weeks. Potent cognitive function‐relevant factors, including the escape latency, time percentage in the goal quadrant, and numbers of CD34^+^ cells, von Willebrand Factor^+^ (vWF ^+^) vessels, and circulating endothelial progenitor cells (EPCs), were subsequently assessed using the Morris water maze (MWM) test, immunohistochemical staining, and flow cytometry.

**Results:**

Compared with the rats in the N‐ES group, the rats in the ES group exhibited a shorter escape latency on day 3 (*p *=* *.025), day 4 (*p *=* *.011), and day 5 (*p *=* *.003), as well as a higher time percentage in the goal quadrant (*p *=* *.025) in the MWM test. After 3 weeks of ES, there were increased numbers of CD34^+^ cells (*p *=* *.008) and vWF ^+^ vessels (*p *=* *.000) in the hippocampus of injured brain tissue in the ES group compared with those in the N‐ES group. Moreover, ES also significantly increased the number of EPCs in the peripheral blood from days 3 to 21 after TBI in the ES group (*p *<* *.05).

**Conclusions:**

Taken together, these findings suggest that ES may improve cognitive deficits induced by TBI, and this protective effect may be a result, in part, of enhanced angiogenesis, which may be attributed to the increased mobilization of EPCs in peripheral blood.

## 1 INTRODUCTION

Traumatic brain injury (TBI) remains a leading cause of disability and death in young individuals in China (Wu et al., 2008). Many patients survive; however, they often suffer from poor long‐term outcomes, such as cognitive deficits, because of a lack of effective treatments (Bohnen, Jolles, & Twijnstra, 1992). In the past several years, numerous therapies have been investigated; however, they have failed to improve TBI‐associated cognitive deficits. Intravenous corticosteroid administration after brain trauma has been ineffective and increased mortality (Edwards et al., 2005). Our previous tests have also indicated that high‐dose glucocorticoids promote hippocampal and hypothalamic neuronal apoptosis, which enhance cognitive deficits after TBI (Chen, Zhang, Yang, Dong, & Zhang, 2009). Hypothermia treatment for TBI has received widespread attention; however, its efficacy in improving outcomes in patients remains to be demonstrated (Chen et al., 2009; Safar & Kochanek, 2001). Thus, there is an urgent need to develop a safe and effective treatment for TBI patients.

Recently, increasing attention has focused on electrical stimulation (ES), a novel therapy in the treatment of diseases associated with vascular injury in both experimental and clinical research. It has been reported that ES in myocardial infarction is not only safe but also effective in promoting angiogenesis (Zhang, Liu, He, Liu, & Feng, 2011). Electrical stimulation may accelerate cutaneous healing by downregulating inflammation, upregulating angiogenesis, and advance remodeling in humans (Sebastian et al., 2011). These therapeutic effects have been confirmed via the phosphoinositide 3‐kinase/Akt signaling pathway in animal experimental studies (Baba et al., 2009). In addition, researchers have reported that ES promotes functional recovery and brain remodeling by enhancing angiogenesis in a rat model of ischemia (Cheng et al., 2012). Similarly, angiogenesis plays an important role in neurological recovery after TBI (Cheng et al., 2012; Huang et al., 2013; Zhang et al., 2013). However, to date, limited studies have investigated the effects of ES treatment on TBI.

In this study, we hypothesized that ES induced an increase in the number of circulating endothelial progenitor cells (EPCs), which contributed to new blood vessel formation in postnatal angiogenesis after TBI and subsequently improved cognitive deficits. We tested this hypothesis in a rat model of experimental TBI.

## 2 MATERIALS AND METHODS

### 2.1 Animals

Adult male Wistar rats (280–320 g; Experimental Animal Laboratories of the Academy of Military Medical Sciences; Beijing, China) were individually housed in a temperature (22°C) and humidity‐controlled (60%) vivarium; they were maintained on a standard 12 hr light/dark cycle (7:00 a.m. to 7:00 p.m. per cycle) with free access to food and water. Experiments were designed to minimize the number of animals required, and the animals used were cared for, handled and medicated appropriately to minimize their suffering. All experimental procedures were approved by the Hospital Animal Care Committee based on guidelines set by the Chinese Small Animal Protection Association.

### 2.2 Experimental groups

The rats were randomly divided into three groups with 28 animals per group: (1) Sham control group: rats underwent the surgical procedure of fluid percussion, without exposure to percussion injury. (2) ES group: rats received ES treatment after fluid percussion‐induced TBI. (3) No electrical stimulation control group (N‐ES group): rats received the same procedure as the ES group, without ES.

### 2.3 Fluid percussion injury model

The fluid percussion injury (FPI) model is a broadly used animal model of closed brain injury (Dixon et al., 1987). Briefly, the rats were anesthetized with 10% chloride hydrate (3.0 ml/kg, intraperitoneal injection) and placed in a stereotaxic frame. The scalp was reflected with a single incision, and the temporal muscles were scraped from the skull. Craniotomy (4.0 × 4.0 mm) was performed over the right parietal skull, 2.0 mm lateral from the sagittal suture and 3.0 mm caudal from the coronal suture, with the dura intact. A luer‐loc connector (3 mm diameter) was subsequently secured to the skull over the opening with cyanoacrylate adhesive and dental acrylic. The skull sutures were sealed with the cyanoacrylate to ensure that the fluid bolus from the injury remained within the cranial cavity. Twenty‐four hours after surgery, the rats were subjected to experimental FPI of 2.0–2.2 atmosphere (atm) using a FPI device (model 01‐B; New Sun Health Products, Cedar Bluff, VA, USA). A rapid bolus of saline from a Plexiglas cylindrical reservoir was introduced into the closed cranial cavity, which caused mechanical deformation of the brain. Immediately after FPI, the incision was suture‐closed; the rats were returned to a heating pad until ambulatory and subsequently returned to their home cage.

### 2.4 Electrical stimulation treatment

Following the generation of the experimental TBI rat model, we immediately applied noninvasive ES as previously described by Ji et al. (1998) to the rats in the ES group for a period of 3 weeks. Briefly, the rats were placed in a plastic immobilizer, and their ears were wiped with saline. Ear clip electrodes were placed on both ears of the rats and were connected to an ES (unit J18A1; Quanrikang, Beijing, China). The ES was administered by passing a current (5 mA at 50 Hz) 30 min daily. This current intensity did not cause an electric burn, convulsion, or other discomfort in the rats. The same procedure of the ES group was performed on the N‐ES group without passing current. The rats were returned to their home cage after these procedures.

### 2.5 Morris water maze task

Learning abilities were assessed using the Morris water maze (MWM) 3 weeks (at day 22–26) after TBI. The rats were trained using the MWM (DMS‐2, Chinese Academy of Science, China) according to the protocol described by Vorhees and Williams (2006). Briefly, a tank that measured 150 cm in diameter by 50 cm in height was filled with water (20–22°C). A target platform (10 cm diameter) was hidden 2 cm below the water surface in a southeast location halfway between the center and the wall of the maze. The rats were allowed to adapt to the maze without a platform for 1 min per day for 3 days prior to training. The rats were subsequently trained to rely on visual distal cues to locate a submerged escape platform. A computerized tracking system (EthoVision XT, RRID:SCR_000441) was used to record the latency (time to reach the platform) and swim speed. Four trials from four random start positions (west, north, southwest, and northeast) were tested daily (each trial lasted 120 s with 15 s intervals) for 5 consecutive days (from 1 day through 5 days post‐ES treatment). The rats that failed to find the platform within 2 min were recorded with a maximum latency score of 120 s. The latency (second) and path length (cm) were recorded over time to generate a spatial learning curve. At 6 day after treatment, we removed the platform and performed a probe trial with a novel start position facing the tank wall. We recorded the time the rats remained in the goal quadrant during a 30 s period. The swim speed (cm/s) was also recorded and used to exclude potential changes that were a result of trauma‐induced movement impairment.

### 2.6 Tissue processing

At different time‐points after TBI, the rats were deeply anesthetized and euthanized via slow transcardiac perfusion with ice‐cold phosphate‐buffered saline (PBS), followed by ice‐cold 4% paraformaldehyde in PBS. Brain areas involved in the traumatic foci were recut from the whole brain and immersed in 4% paraformaldehyde overnight, followed by cryoprotection in 30% sucrose‐containing PBS. Coronal sections of 8‐μm thickness were sectioned from each sample and stored at −80°C for further analysis.

### 2.7 Immunohistochemistry staining

CD34^+^ cells in brain tissue were detected using a CD34 antibody (R and D Systems Cat# AF4117 Lot# RRID:AB_2074613) as recommended by the manufacturer. Briefly, after deparaffinization and redehydration, the nonspecific endogenous peroxidase activity was blocked by treating the sections with 3% hydrogen peroxide in methanol for 30 min. Antigen recovery was performed by boiling the sections for 20 min in 10 mmol/L citrate buffer (pH 6.0). The nonspecific binding was blocked with 3% bovine serum albumin (BSA) in PBS for 30 min. The sections were subsequently incubated with a goat anti‐rat CD34 antibody (1:200) overnight at 4°C. The sections were washed with PBS, incubated with a biotinylated anti‐goat IgG (Zhongshan, Beijing, China) for 1 hr at 37°C, and rewashed and incubated in an avidin peroxidase conjugate solution (Zhongshan) for 30 min. Finally, the sections were developed with diaminobenzidine for 3 min. Negative controls were similarly processed in the absence of primary antibody. The number of endothelial‐like CD34^+^ cells in each section was quantified (per 200×; Olympus‐IX2UCB, Tokyo, Japan) in five fields by two independent observers who were blind to the experimental conditions to obtain an average number of CD34^+^ cells per viewing field.

The microvasculature was quantified via the quantification of von Willebrand Factor^+^ (vWF^+^) vessels (Abcam Cat# ab6994 Lot# RRID:AB_305689) using a protocol similar to the CD34 staining protocol. Five sections were quantified under a light microscope (per 200×; Olympus‐IX2UCB, Tokyo, Japan) by two independent observers who were blind to the experimental conditions. A brown stained vessel‐lumen structure was defined as a vessel.

The sections were sequentially selected and stained with CD34 and vWF.

### 2.8 Measurement of EPCs via flow cytometry

Peripheral blood samples (0.5 ml) were collected from the retro‐orbital venous plexus at baseline (0) 1, 3, 7, 14, 21, and 28 days after TBI and diluted with PBS. Peripheral blood mononuclear cells were isolated via density‐gradient centrifugation using Ficoll‐Paque Plus (Chuanye, Tianjin, China). The isolated cells were washed twice with PBS and resuspended in 200 μl of PBS supplemented with 0.5% of BSA and 2 mmol/L of EDTA. EPCs in the peripheral blood were evaluated by staining with PE‐conjugated CD34 monoclonal antibody (Santa Cruz Biotechnology, Santa Cruz, CA, USA) and purified CD133 primary antibody (Abcam Cat# ab19898 Lot# RRID:AB_470302) conjugated with FITC (Abcam Cat# ab6717 Lot# RRID:AB_955238), followed by the detection via flow cytometry (HMS NERCE FACSCalibur Flow Cytometer Resource, RRID:SCR_000879). The isotype‐matched IgG was used as a control.

### 2.9 Statistical analysis

Statistical analysis was performed using SPSS16.0 (SPSS, RRID:SCR_002865). The data are presented as the mean ± standard error of the mean. Repeated measures ANOVA with the post hoc LSD test were used to compare the cognitive function tested by the MWM. One‐way ANOVA with the post hoc LSD test were used for the analysis of the CD34^+^ cells, vWF^+^ vessels and circulating EPCs in each group. A *p*‐value <.05 was considered statistically significant.

## 3 RESULTS

### 3.1 Electric stimulation treatment improved cognitive deficits induced by TBI

**Figure 1:**
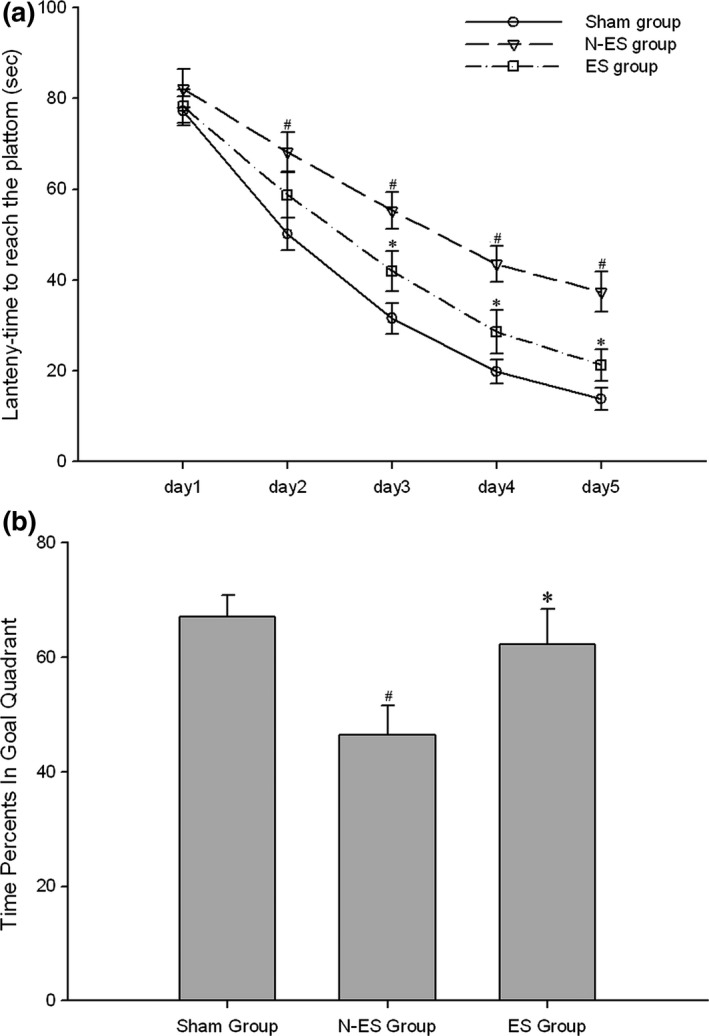
Electrical stimulation (ES) treatment significantly improved the cognitive deficit associated with traumatic brain injury (TBI) by Morris water maze tests. (a) This data suggested that the escape latency of all rats was influenced by grouping. The test revealed that the escape latency in Sham group was significantly shorter than that in the N‐ES group from day 2 to 5. And there was no difference between Sham group and ES group. Furthermore, the escape latency was significantly decreased in ES group compared to N‐ES group from day 3 to 5. (b) Six days after training, the platform was removed and the ability of rats to find the removed platform through spatial memory was measured as percent of times they swam in the goal quadrant. Compared with the N‐ES group, rats in the ES group had significantly increased time percents in goal quadrant. However, there was no difference to the percents between ES group and Sham group. *n* = 10/group; *p < .05 ES group versus N‐ES group; ^#^ *p* < .05 N‐ES group and ES group versus Sham group

To assess cognitive function changes, the spatial memory of the rats was examined in the MWM. Expectedly, the latency was significantly shortened during the 5‐day spatial acquisition test, which suggested that spatial memory was developed in all rats (*p *=* *.000, Figure 1a). However, the escape latency of all rats was affected by grouping (*p *=* *.001, Figure 1a). The test indicated that the escape latency in the Sham group was significantly shorter than the escape latency in the N‐ES group on day 2 (*p *=* *.006), day 3 (*p *=* *.000), day 4 (*p *=* *.000), and day 5 (*p *=* *.000). There was no difference between the Sham and ES groups. Compared with the rats in the N‐ES group, the rats in the ES group had a shorter latency at day 3 (*p *=* *.025), day 4 (*p *=* *.011), and day 5 (*p *=* *.003), which indicated a better recovery of cognitive functions after TBI.

On day 6 after training, the platform was removed, and the rats were tested for their ability to look for the removed platform using spatial memory, which was measured as the percentage of times they swam in the goal quadrant (reference memory). Compared with the N‐ES group, the rats in the ES group demonstrated a significantly increased percentage of time in the goal quadrant (*p *=* *.025, Figure 1b). However, there was no difference in the percentage of time between the ES and Sham groups (*p *=* *.475, Figure 1b). There were no group differences in the swimming speed of the injured rats before and after injury.

### 3.2 Electrical stimulation treatment promoted angiogenesis in the hippocampus

**Figure 2:**
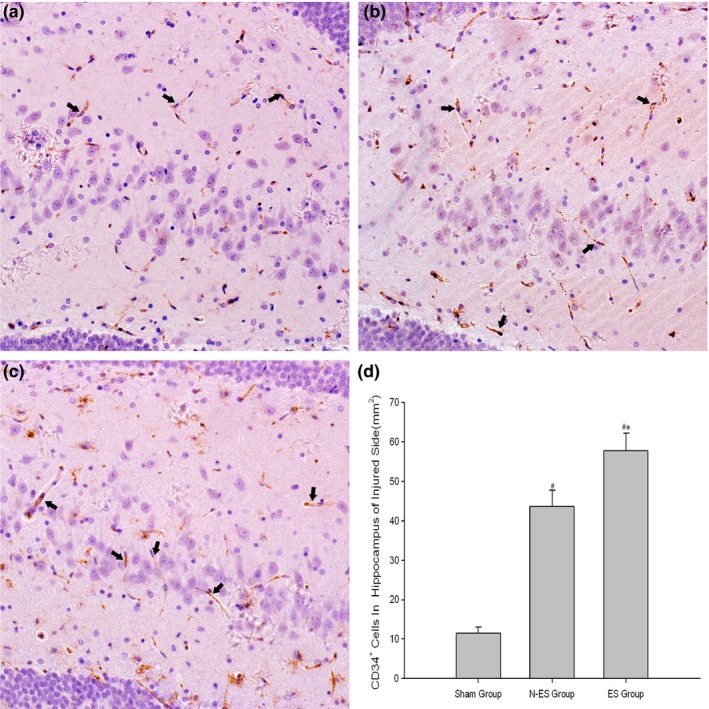
Detection of CD34^+^ cells in the CA1 region, a subdivision of Ammon's horn of hippocampus, of the injured side after electrical stimulation treatment by immunohistochemistry staining. (a) Sham group, (b) no electrical stimulation(N‐ES) group, (c) electrical stimulation (ES) group, (d) quantitative data of CD34 positive cells. The classical CD34^+^ cells are directed by black arrow. They were defined as the cells which are spindle‐shape and brown staining. *n* = 6/group; *p < .05 ES group versus N‐ES group; ^#^ *p* < .05 N‐ES group and ES group versus Sham group

CD34 is a marker for progenitor hematopoietic cells and is expressed on sprouting microvascular endothelial cells. Thus, CD34 positivity indicates angiogenesis that stems from hematopoietic progenitor cells. In this study, we identified more CD34^+^ cells in the hippocampus of the rats in the N‐ES and ES groups than in the Sham group at 3 weeks after ES; these findings indicate that CD34^+^ EPCs were mobilized from the bone marrow and recruited into the injured brain. In addition, we identified higher levels of CD34^+^ cells in the ES group than the N‐ES group (*p *=* *.008, Figure 2).

**Figure 3:**
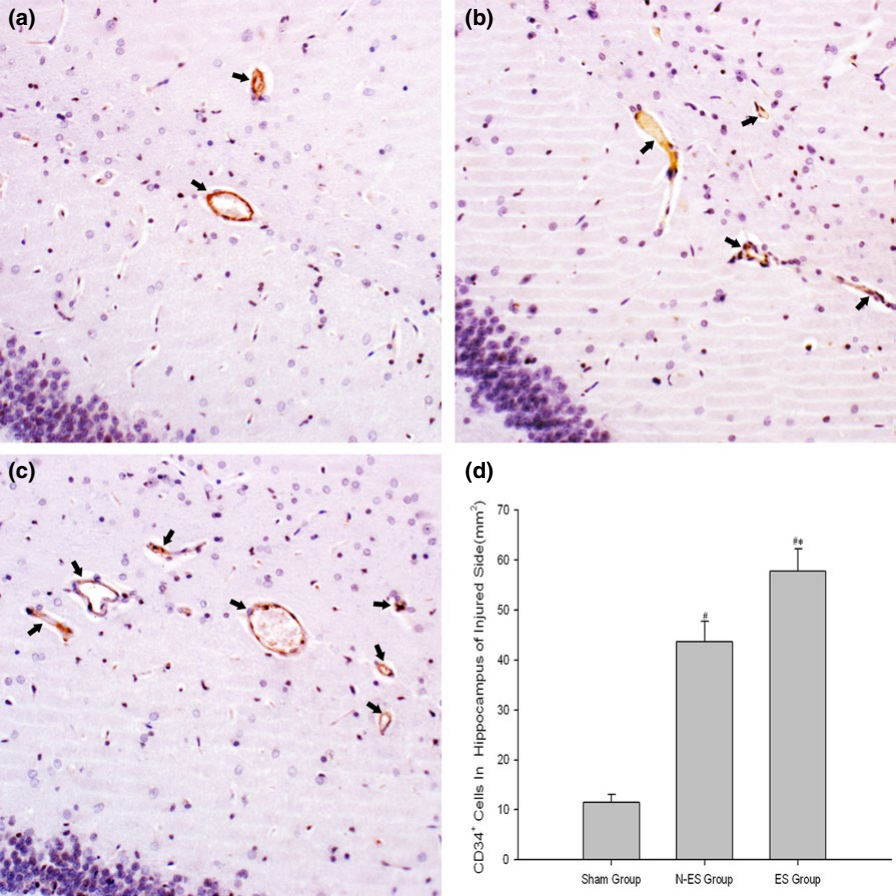
Detection of von Willebrand Factor (VWF)^+^ vessels in the CA1 region, a subdivision of Ammon's horn of hippocampus, of the injured side after electrical stimulation treatment by immunohistochemistry staining. (a) Sham group, (b) NES group, (c) ES group, (d) quantitative data of VWF positive vessels. The classical VWF ^+^ vessels are directed by black arrow, and which have the brown staining lumina formation. *n* = 6/group; *p < .05 ES group versus NES group; ^#^ *p* < .05 N‐ES group and ES group versus Sham group

We labeled the endothelial cells with vWF, a commonly used vascular marker, to detect vessel changes between the groups. The number of vWF^+^ vascular cells in the rats that suffered from TBI was significantly increased at 3 weeks post injury. In addition, there were more vWF^+^ vascular cells in the hippocampus of the injured side in the ES group than in the N‐ES group (*p *=* *.000, Figure 3).

### 3.3 Electrical stimulation increased the number of EPCs in peripheral blood

**Figure 4:**
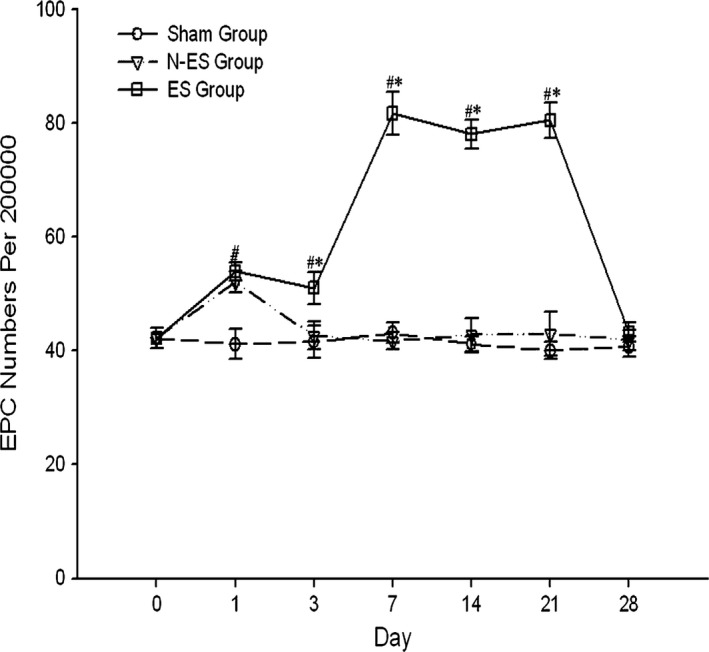
Flow cytometry detection of endothelial progenitor cells (EPCs) in peripheral blood of rats before the traumatic brain injury (TBI) (0 day), and 1, 3, 7, 14, 21, 28 days after TBI. They were marked by CD34 and CD133. The stress of TBI mobilized the EPCs at 1 day after TBI in N‐ES group and ES group. Electrical stimulation treatment increased EPCs numbers in peripheral blood from 3 to 21 days after TBI. *n* = 6/group; *p < .05 ES group versus N‐ES group; ^#^ *p* < .05 N‐ES group and ES group versus Sham group

We measured the number of EPCs colabeled with CD34 and CD133 using flow cytometry to detect alterations. The rats in the Sham group served as controls. Peripheral blood samples were collected before TBI (0 day) and at 1, 3, 7, 14, 21, and 28 days after TBI. Figure 4 indicates that EPCs were upregulated on day 1 after TBI and subsequently returned to baseline in the N‐ES group, whereas the EPC levels of the ES group increased from days 3 to 21 after TBI. This high expression returned to baseline on day 28 (the ES treatment was terminated on day 21 after TBI).

## 4 DISCUSSION

In this study, we examined cognitive deficits in rats subjected to TBI and correlated changes in the number of CD34^+^ cells and vWF^+^ vascular cells in the hippocampus of injured brain tissue and EPCs in the peripheral blood. We confirmed that ES improved the cognitive deficits associated with TBI in rats. In addition, ES shortened the escape latency and increased the percentage of time spent in the goal quadrant during the MWM test. Electrical stimulation also increased the number of CD34^+^ cells and vWF^+^ vascular cells in the injured hippocampus, which was correlated with vascular remodeling after brain trauma. Electrical stimulation further increased the number of EPCs in the peripheral blood. Our previous study confirmed that these cells could home to injured brain tissue and promote angiogenesis (Zhang et al., 2013).

Cognitive deficits represent common neurological damage in patients with TBI (Franulic, Horta, Maturana, Scherpenisse, & Carbonell, 2000). To date, there are no effective therapies to prevent TBI (Franulic et al., 2000; Twamley, Jak, Delis, Bondi, & Lohr, 2014). In this study, we determined that ES could improve cognitive deficits after TBI. The current method is noninvasive. It is more convenient and economical and exhibits a lower infection rate compared with invasive approaches, such as deep brain stimulation, vagus nerve ES, and cortical ES. Moreover, it plays a similar protective role in central nervous system diseases as invasive methods.

The recovery processes of brain function after TBI include angiogenesis, neurogenesis, and synapse formation. Of these processes, angiogenesis is the most important and essential. It not only provides the nutrients required for neurogenesis and synapse formation but is also conducive in the clearance of cytotoxic substances (Ning et al., 2011). The number of CD34^+^ cells and vWF^+^ vascular cells in the injured hippocampus after ES treatment was increased in this study. Both of these cell types are closely correlated with angiogenesis. CD34^+^ cells are progenitor hematopoietic cells, which can differentiate into mature endothelial cells and secrete angiogenic factors (Guo et al., 2009). Campagnolo et al. (2010) determined that CD34^+^ cells isolated from the saphenous vein promoted angiogenesis and improved blood flow in animal models of an ischemic limb. vWF is a commonly used marker for vessels. Our previous study confirmed that an increased number of vWF^+^ vascular cells in brain tissue was correlated with a better outcome of TBI in rats (Wang et al., 2012).

Endothelial progenitor cells are related to hemangioblast stem cells and contribute to new blood vessel formation in postnatal vasculogenesis and angiogenesis (Isner & Asahara, 1999). With specific stimuli, they may be mobilized from bone morrow to peripheral blood and subsequently recruited into the angiogenic niche of injured tissue (Schier et al., 2014). The administration of erythropoietin increases EPC mobilization and promotes vascular remodeling, as well as improves neurological outcome after stroke (Pellegrini et al., 2013). Consistent with these findings, studies have also indicated a positive correlation between the number of EPCs and the clinical outcomes of patients with TBI (Liu et al., 2011). Moreover, our previous study showed that the administration of progesterone increased the number of circulating EPCs and induced neural regeneration after TBI in aged rats (Li et al., 2012). Furthermore, TBI rats treated with atorvastatin exhibited better neurological outcomes with an increasing number of EPCs (Li et al., 2012; Wang et al., 2012). In this study, we determined that the level of EPCs was significantly increased in the peripheral blood with ES, and better cognitive function was identified in the same group. The potential mechanisms of these EPC mobilizations may involve several factors. Stromal cell‐derived factor‐1 (SDF‐1) is a primary factor that contributes to EPC mobilization by binding to CXCR4 expressed on circulating cells. Vascular endothelial growth factor A (VEGF‐A) enhances EPC recruitment and homing via its engagement with VEGFR‐1 and VEGFR‐2 on EPCs. SDF‐1 and VEGF also mutually promote mobilization (Jin et al., 2006; Li et al., 2006; Petit, Jin, & Rafii, 2007; Zhang et al., 2013). Electrical stimulation may, at least in part, induce factors involved in these pathways to promote EPC homing after TBI, which requires further investigation.

In summary, the effects of ES on neurological damage induced by brain injury have been investigated for many years. Electrical stimulation may improve neurological outcome by evoking dopaminergic release in the striatum or increase 5‐hydroxytryptamine expression in the subventricular zone (Gale et al., 2013; Jahanshahi et al., 2013). The current findings suggest an additional important pathway, EPC mobilization and vascular repair, in this process. Electrical stimulation increases the number of circulating EPCs and promotes angiogenesis in the injured hippocampus, which may contribute to the improvement of cognitive deficits after TBI.

## CONFLICT OF INTEREST

The authors declare there is no conflict of interest.
